# Portable bedside ultrasound: the visual stethoscope of the 21^st ^century

**DOI:** 10.1186/1757-7241-20-18

**Published:** 2012-03-09

**Authors:** Lawrence M Gillman, Andrew W Kirkpatrick

**Affiliations:** 1 Department of Surgery, University of Manitoba, Z3053 - 409 Tache Avenue, Winnipeg, Manitoba, R2H 2A6, Canada; 2 Departments of Surgery, Critical Care Medicine, and Regional Trauma Services, University of Calgary, Calgary, Alberta, Canada

**Keywords:** Point of care ultrasound, Physical exam, Pleural rub

## Abstract

Over the past decade technological advances in the realm of ultrasound have allowed what was once a cumbersome and large machine to become essentially hand-held. This coupled with a greater understanding of lung sonography has revolutionized our bedside assessment of patients. Using ultrasound not as a diagnostic test, but instead as a component of the physical exam, may allow it to become the stethoscope of the 21^st ^century.

## Introduction

Until recently, the role of ultrasound has most notably been as an anatomic imaging test, confined to a specialized department in specialized hands. In the past decade, as technology continues to improve, ultrasound has made a move from the department to the bedside. First introduced in the emergency department, ultrasound was initially used as an adjunct in the trauma bay for the detection of pericardial or free intra-abdominal fluid. Its role has since expanded to the diagnosis of basic intra-abdominal pathology, safer line and tube insertion and confirming pregnancy, among many other uses.

For generations the stethoscope has been a symbol of the medical profession. Early physicians had only archaic tools at their disposal and while many have fallen by the wayside, the stethoscope remains an important part of medical culture. However, spawned by recent technological advances, there is now a movement afoot to use ultrasound as ubiquitously as the stethoscope. Ultrasound and the stethoscope share many similarities. Both are operator dependent, requiring practice and expertise to develop appropriate technique and skill. Comparing auscultation to echocardiography there is no question as to the superiority of ultrasound in the right hands, however pulmonary examination has been the limiting factor ... until recently.

Because ultrasound waves are nearly completely reflected by an air tissue interface, it was originally concluded that "ultrasound imaging is not useful for evaluation of the pulmonary parenchyma" [1]. However, recent evidence reveals that an examination of the artifacts produced by lung ultrasound results in a wealth of information about the underlying lung.

## Methods

A review of the medical literature was performed using Pubmed and articles pertaining to ultrasound-assisted assessment of the lung and the pleural space. The references of these articles were reviewed in order to locate additional articles. The literature selected was based on the preference and clinical expertise of authors.

## Discussion

### Equipment and technique

Pleural and lung ultrasound can be performed with a number of probe types including high frequency linear probes or lower frequency curvilinear probes and cardiac phased arrays. A potentially contentious issue in lung ultrasound literature regards the appropriate probe choice. As this modality is still in its infancy and industry has yet to develop a dedicated lung transducer there is still not an accepted probe for this procedure and thus studies vary considerably in the probe used. The most popular probe seem to be a microconvex one ranging in frequency between 2-5 MHz [2-6]. However, others have used high frequency linear transducers (5-10 MHz) [7-9] or cardiac phased arrays (2-4 MHz) [10,11]. No particular ultrasound machine is required and moderately priced portable machines will perform quite well. Useful adjuncts that aid certain diagnoses are M-mode (or time-motion mode) and colour power doppler.

To begin a pulmonary exam, the probe is applied tangentially to the chest wall and is oriented longitudinally. The "pleural line", which represents the parietal and visceral pleural interface, can typically be seen 0.5 cm below and between two rib shadows. Together, the upper rib, pleural line, and lower rib form a characteristic pattern, the bat sign [12].

### Important artifacts

**Figure 1:**
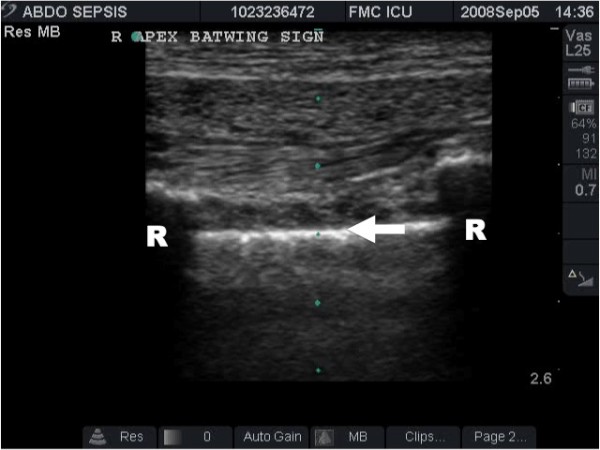
**The hallmark of lung ultrasound illustrating the normal lung**. The pleural line (arrow) is seen below the rib shadows (R) on either side. In real time ultrasound, lung sliding - the visual equivalent of breath sounds, can be seen as motion at the pleural line.

The hallmark of lung ultrasound is lung sliding (Figure 1, Additional file 1). This refers to a to-and-fro movement of the mobile visceral pleural along the static parietal pleura that is synchronized with respiration [9,12,13]. When one becomes familiar with pleural and lung ultrasound, recognizing sliding becomes as reassuring as hearing breath sounds during auscultation.

**Figure 2:**
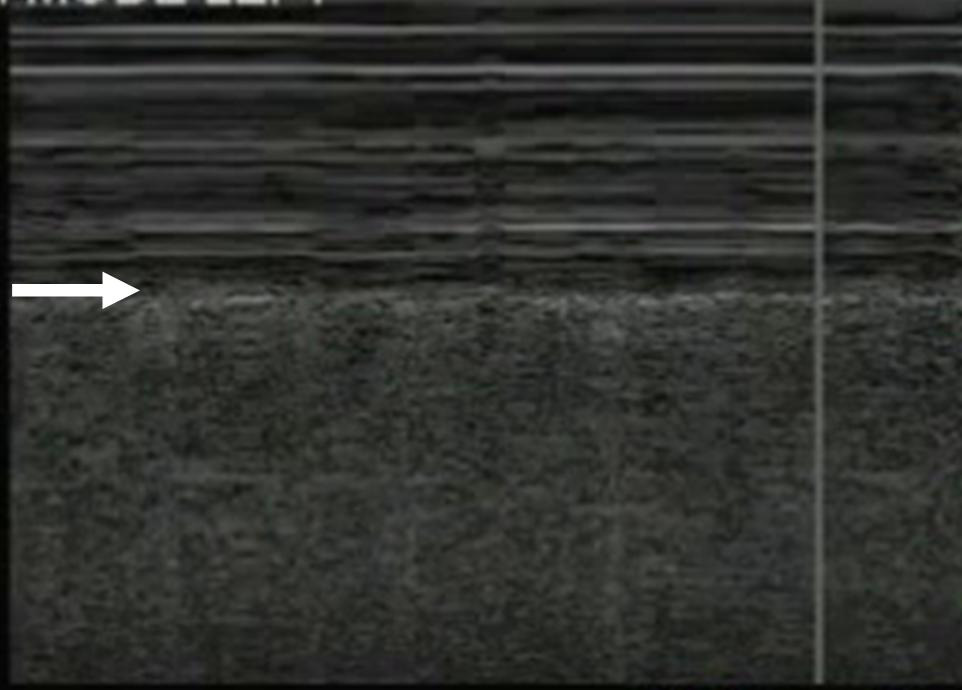
**Time-motion (M-) mode ultrasonography illustrating lung sliding by the presence of the 'seashore sign'**. The 'seashore sign' is characterized by motionless parietal tissue over the pleural line (arrow) and a homogenous granular pattern below it.

**Figure 3:**
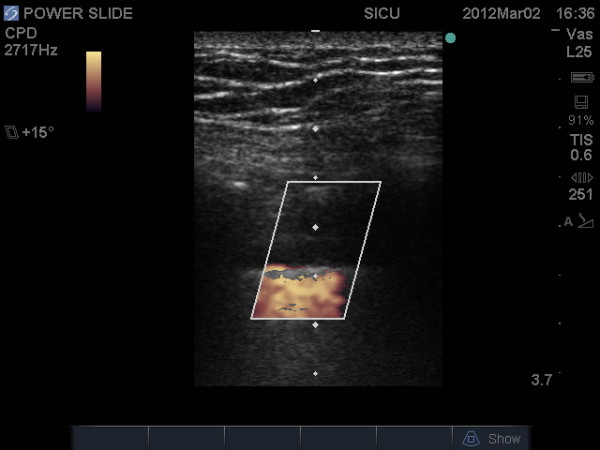
**The "power slide" - Power color doppler image indicating motion at the pleural line confirming the presence of lung sliding**.

The M-mode, or time-motion mode function, can help confirm and document the presence of lung sliding. Time-motion mode is a common ultrasound function that takes a single thin segment of the ultrasound field and displays changes within that segment over time. Motionless segments can be seen as a series of parallel lines, while disruption or obliteration of these parallel lines implies motion. If lung sliding is present then a 'seashore sign' will be present on M-mode. The 'seashore sign' is characterized by motionless parietal tissue over the pleural line and a homogenous granular pattern below it, thus indicating the presence of lung sliding (Figure 2) [12,14]. More recently power color doppler has also been described to help confirm the presence of lung sliding, also called the 'power slide' sign (Figure 3, Additional File 2) [7].

**Figure 4:**
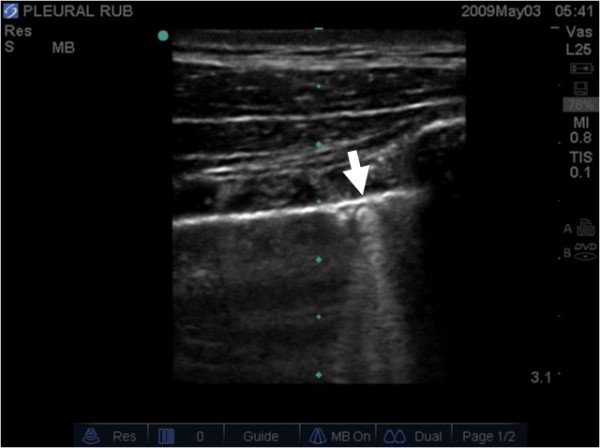
**Lung ultrasound image illustrating comet tail artifacts (arrow) caused by thickening of the interlobular septa**.

A second important artifact is the comet tail artifact. The comet tail artifact, also known as a B-line or lung rocket, was first described in 1982 [15]. It is a vertical narrow-based artifact that spreads out to the edge of the screen (Figure 4, Additional File 3). The comet-tail artifact appears when there is a marked difference in acoustic impedance between an object and its surroundings [15]. These artifacts have been found to correspond to sub-pleural interlobular septa [2] that are generally visible when thickened by edema however, in the normal lung a few scant comet tail artifacts can be seen especially in the lower lobe just above the diaphragm [2].

By interpreting alterations in these important artifacts one can make important inferences about the underlying lung parenchyma. These diagnoses include pneumothoracies, pulmonary edema, pleural effusions and atelectasis/consolidation.

### Pneumothorax

The diagnosis of pneumothorax was a relatively early clinical application of lung ultrasound. In fact, the first description of the role of ultrasound in the diagnosis of pneumothorax dates back to 1986 and was given by a veterinarian in the study of horses [16]. It was described for use in humans one year later [17]. Since then many studies have confirmed that ultrasound offers a highly accurate and rapid method to rule out the presence of a pneumothorax. The sensitivity and specificity reported in the literature may range from 80 - 98% and 94 to 99% respectively [3,10,13,18-21] although readers should be suspicious of studies that use plain radiographs as the gold standard [10,20]. Recent studies reveal that small penumothoracies can be initially missed in 30 to 80% of patients on supine chest radiograph [19,21,22]. CT scan may offer a much more sensitive diagnosis but it requires the patient be transported and significantly delays time to diagnosis.

Air impedes the passage of ultrasound waves, and air contained within the lung and within the pleural space appears similarly on ultrasound. Therefore, much of the work in ultrasound has focused on not confirming the presence of a pneumothorax but instead ruling out a pneumothorax. The presence of lung sliding implies that the visceral and parietal pleura are in apposition and are not separated by air thus precluding the possibility of a pneumothorax. The presence of this sign has been reported to be highly sensitive and specific in ruling out a pneumothorax when seen throughout the lung fields [7,23-25].

The additional finding of a comet-tail artifact also implies that the intra-lobular septa can been seen. This would be impossible if a pneumothorax was present, as the air would interfere with visualization of the underlying lung. Comet-tail artifacts have been shown to rule out a pneumothorax with a negative predictive value approaching 100% [10,26].

There are unfortunately limitations to ultrasound in the diagnosis of pneumothorax. Absence of lung sliding can create false-positive results in certain clinical situations. The presence of bullous emphysema, pleura adhesions and even simple apnea has been associated with loss of the sliding lung sign when no pneumothorax is present [19]. Extensive subcutaneous emphysema can be confused, in novice hands, with the absence of lung sliding, but this should be avoided if the ribs and pleural line (bat wing sign) are clearly identified prior to looking for the pleural interface [12]. However, extensive subcutaneous emphysema can make visualization of the underlying pleura impossible, thus making the study non-diagnostic for both thoracic and abdominal pathologies. It is important to recognize that such studies are neither positive nor negative studies but are formally indeterminate [27].

**Figure 5:**
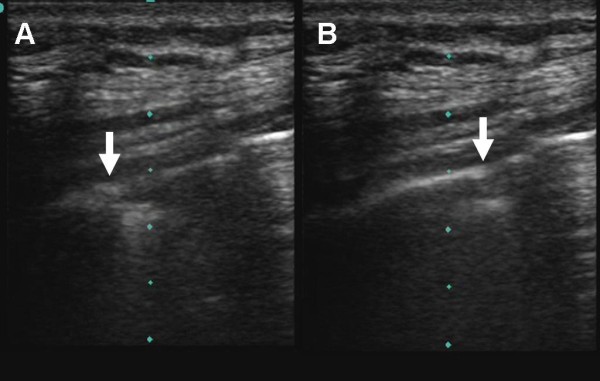
**Normal pleura can be seen on the left side of the image (A) and the lung point (arrow) can be seen moving across the screen with respiration (B)**.

Once the presence of a pneumothorax is suspected the ultrasound examination should then be extended laterally in an attempt to localize the point where the normal lung pattern (lung sliding and/or the presence of vertical B lines) replaces the pneumothorax pattern (absent lung sliding and absent B lines). This point is called the 'lung point' (Figure 5, Additional File 4) and marks the intersection where the lung is re-approximated back with the pleura at the edge of the pneumothorax. The ability to demonstrate the alternating lung sliding and absence of lung sliding within the same ultrasound field has been touted as being 100% diagnostic of a pneumothorax [14].

### Consolidation and atelectasis

While the ultrasound beam cannot penetrate the normal aerated lung, in the presence of alveolar consolidation the lung becomes filled with purulent, water-rich material that is an excellent transmitter of ultrasound waves [28].

**Figure 6:**
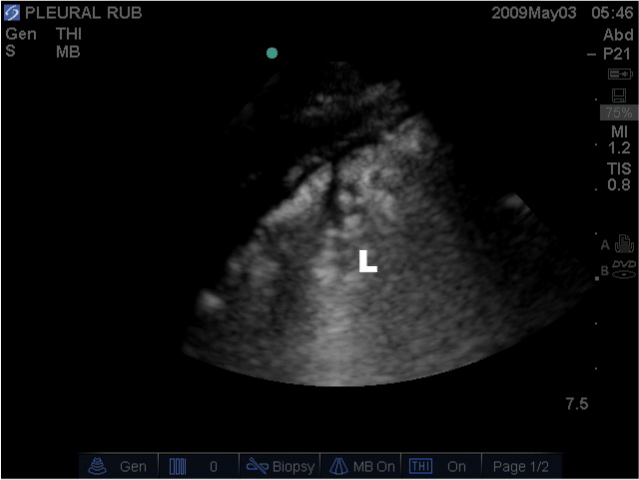
**Lung ultrasound image illustrating lung consolidation, highlighted by lung (L) hepatisation ("appearing liver like")**.

The presence of consolidation produces a tissue-like pattern reminiscent of the liver that has been referred to as 'hepatization' of the lung (Figure 6, Additional File 5) [28]. In addition, internal hyperechoic punctiform or linear elements are frequently seen [28]. These correspond to air within the more rigid, non-collapsed bronchi and are the ultrasound equivalent of the air bronchogram on a chest radiograph.

Limitations to the ultrasound diagnosis of consolidation include the fact that while most consolidation reaches the pleural surface of the lung, areas confined to the interior of the lung, and not reaching the pleural surface, cannot be visualized through overlying aerated lung.

### Alveolar-interstitial syndrome

**Figure 7:**
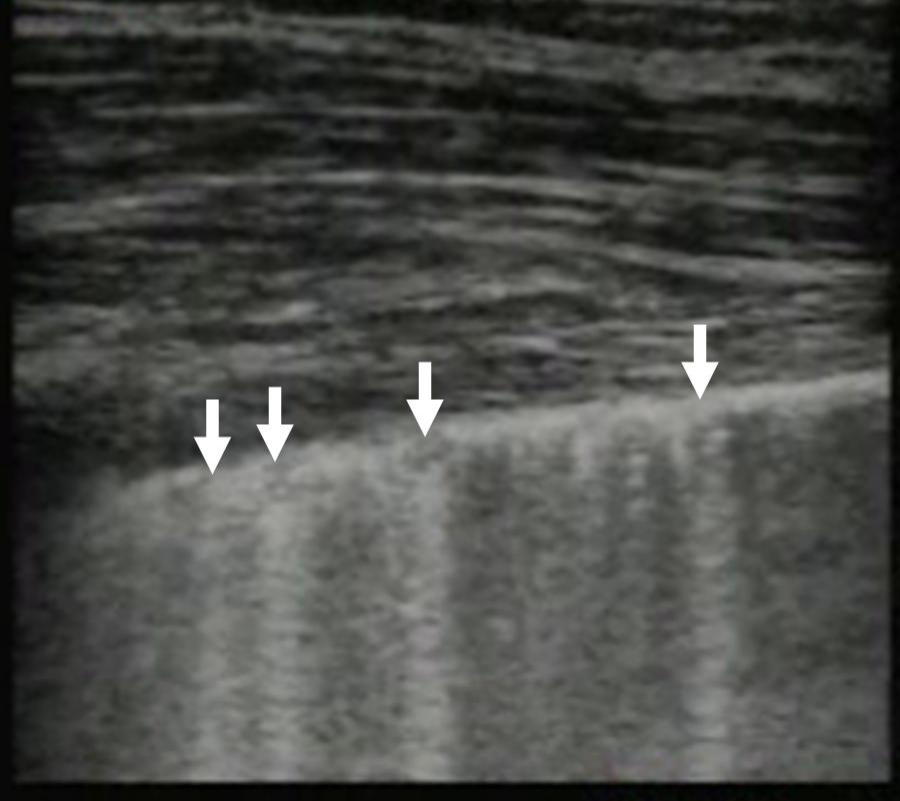
**Image of the lung illustrating multiple comet tail artifacts (arrows) consistent with alveolar-interstitial syndrome, in this case caused by a pulmonary contusion**.

The alveolar-interstitial syndrome includes a group of heterogeneous conditions that are united in that they cause diffuse involvement of the lung interstitium and present a distinct pattern on lung ultrasound [4]. Included in this syndrome are chronic conditions such as pulmonary fibrosis and acute conditions including acute respiratory distress syndrome (ARDS), acute pulmonary edema, and interstitial pneumonia. This diagnosis is made sonographically by noting the presence of multiple comet tail artifacts (Figure 7, Additional File 6). Thus the comet tail artifact is the sonographic equivalent of Kerley's B lines as is seen in pulmonary edema. In fact, a number of studies demonstrated correlation between wedge pressure and extra vascular lung water and the number of comet-tail artifacts [29-33]. However, just as the chest radiograph cannot distinguish between increased interstitial marking from disorders of hydrostatic pressure or permeability, neither can the ultrasound. Noncardiogenic pulmonary edema (such as high altitude pulmonary edema) appears similar to cardiogenic pulmonary edema even though the pulmonary artery wedge pressure is generally normal or low [34].

A small number of B-lines, especially at the bases can be considered normal. One study suggested the presence of more than 3 B-lines per field as criteria for a pathologic number of B-lines [2] while other authors considered a minimum number of six artifacts. However, these studies were conducted with different probes; the first, a microconvex probe and the latter, a linear probe [8].

It should also be noted that B-lines can also be seen isolated in areas surrounding alveolar consolidations of any etiology [5] making it important to differentiate between diffuse B-lines in all lung fields as seen in diffuse alveolar-interstitial syndromes such as pulmonary edema, and the localized B-lines seen in pneumonia or contusion.

### Pleural effusions

**Figure 8:**
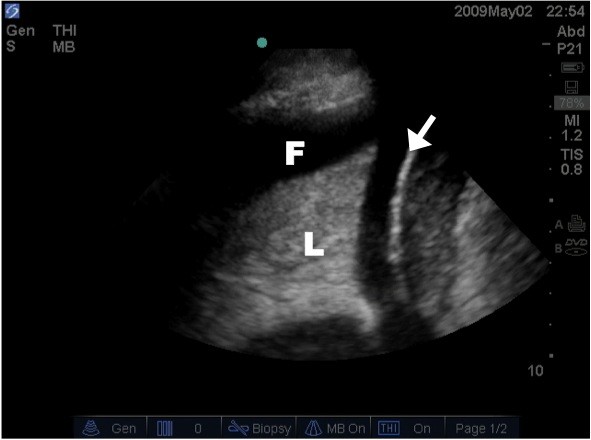
**Lung ultrasound image illustrating the presence of a pleural effusion (P) around the atelectatic lung (L) above the diaphragm (arrow)**.

Quantifying the size of the effusion is often challenging using physical exam and chest radiography. Lobar collapse or atelectasis can often be difficult to differentiate from effusion on chest radiograph and in this case blind aspiration can result in significant complications including hemorrhage and pneumothorax if the diagnosis is incorrect. The supine chest radiograph was found to have a sensitivity of only 39% and an accuracy of 47% in detecting pleural effusion [6]. In multiple studies ultrasound has been shown to be superior than bedside chest x-ray both in diagnosis of pleural effusion and quantification of fluid volume (Figure 8, Additional File 7) [35,36]. Studies have shown that measurements of the distance between the parietal and visceral pleura at end expiration in the ventilated patient at the level of the diaphragm correlate with pleural effusion size [36]. Ultrasound can also help rule out other etiologies such as consolidation, mass, or an elevated hemidiaphragm. In addition, bedside ultrasound can simultaneously help aid in safe, image guided drainage of the pleural effusion [37].

### Ultrasound as a part of the physical exam and its limitations

As illustrated by the preceding literature review most studies of lung ultrasound to date use either chest xray or CT scan as a gold standard. In fact to our knowledge only a single study looks at a comparison between lung ultrasound and auscultation [6]. It is our opinion that this is a huge limitation of the current literature. We do not advocate that bedside ultrasound should replace definitive diagnostic testing but rather should be seen as a part of the physical exam. However, in many of the preceding studies referenced ultrasound is shown to be superior to supine chest xray, especially in the diagnosis of occult pneumothoracies [3,9,18,38-40] and pleural effusions [35,36], and in these instances, in our opinion, one could infer that ultrasound would be superior to auscultation as well.

As a part of the physical exam the sky is the limit in terms of applications of lung ultrasound. We encourage its use wherever a stethoscope is used, making it an essential tool not only in the hospital setting, but also in the office and in the field. In addition, lung ultrasound can be performed with bedside ultrasound units already available in most emergency departments, intensive cares, wards and even in some primary care offices. Further, while auscultation is generally a personal experience, the ultrasound produces a visual image that can be shared simultaneously by many, even remotely [41], and can preserve images in a digital format allowing for accurate documentation and medical education. While generally better supported, this is not entirely unique to ultrasound as some newer stethoscopes do include the possibilities of recording and storage.

The main limitation we foresee with bedside ultrasound comes not from its use, but its misuse. We stress that bedside ultrasound should serve to guide clinical decision making, ideally confirming pre-examination clinical suspicions, but not be performed in lieu of further confirmatory diagnostic testing.

Excessive sensitivity also presents a challenge. In the setting of trauma, the literature reveals that bedside ultrasound has sensitivity for pneumothorax that far exceeds the sensitivity of a chest xray [3,9,18,38-40]. As a result, small, formerly "occult" pneumothoracies are now being discovered which previously may have gone untreated, with no detriment to the patient [42]. Using ultrasound to quantify the size of the pneumothorax may help avoid unnecessary interventions [3].

The major diagnostic limitation of ultrasound compared to auscultation is in the diagnosis of asthma and chronic obstructive pulmonary disease. These obstructive diagnoses reveal only a normal ultrasound pattern with normal lung sliding [43]. However, in the patient with acute respiratory distress, ultrasound has been used with high sensitivity and specificity to rule out other diagnoses, especially pulmonary edema [44,45].

Cost is also currently a relative limitation to its use. While the cost of even handheld units far exceeds of the cost of a stethoscope, as technology continues to evolve portable units will become cheaper and more robust.

A final criticism of bedside ultrasound is the over-reliance on imaging to the detriment of physical exam skills. Again, we would argue that bedside ultrasound not be seen as an alternative to physical exam but instead as a part of the physical exam and hopefully will eventually be taught in medical schools alongside physical exam skills.

## Conclusions

As newer generations of clinicians incorporate it as a part of their basic bedside examination and as technological advances make ultrasound less and less prohibitive in terms of cost and size, we argue its time to augment the archaic tools of past centuries and embrace ultrasound as the visual stethoscope of the 21^st ^century.

## Competing interests

The authors of this paper have nothing to disclose and received no funding for this publication.

## Authors' contributions

LG and AK both drafted the manuscript, were involved in image acquisition and editing, and read and approved the final manuscript.

## Supplementary Material

Additional file 1 **Normal Lung Sliding**. The hallmark of lung ultrasound illustrating the normal lung. The pleural line is seen below the rib shadows on either side. Lung sliding, the visual equivalent of breath sounds, can be seen as motion at the pleural line. Click here for file

Additional file 2 **Power Slide**. The "power slide" - Color doppler image indicating motion at the pleural line confirming the presence of lung sliding. Click here for file

Additional file 3 **Comet Tail Artifacts**. Real time lung ultrasound video illustrating comet tail artifacts caused by thickening of the interlobular septa. Click here for file

Additional file 4 **Lung Point**. Normal pleura can be seen on the left side of the image and the lung point can be seen moving across the screen with respiration. Click here for file

Additional file 5 **Lung Consolidation**. Real time lung ultrasound video illustrating lung consolidation, highlighted by hepatisation of the lung (lung tissue appears similar density to the liver). Click here for file

Additional file 6 **Alveolar-Interstitial Syndrome**. Real time lung ultrasound video illustrating multiple comet tail artifacts consistent with alveolar-interstitial syndrome, in this case caused by a pulmonary contusion. Click here for file

Additional file 7 **Pleural Fluid**. Real time lung ultrasound video illustrating the presence of a hypo-echoic pleural effusion seen above the diaphragm. Click here for file
